# Staged Treatment of Posttraumatic Tibial Osteomyelitis with Rib Graft and Serratus Anterior Muscle Autografts—Case Report

**DOI:** 10.3390/jpm13121651

**Published:** 2023-11-27

**Authors:** Bogdan Anglitoiu, Ahmed Abu-Awwad, Jenel-Marain Patrascu, Simona-Alina Abu-Awwad, Anca Raluca Dinu, Alina-Daniela Totorean, Dan Cojocaru, Mihai-Alexandru Sandesc

**Affiliations:** 1 Department XV—Discipline of Orthopedics—Traumatology, Victor Babes University of Medicine and Pharmacy, Eftimie Murgu Square, No. 2, 300041 Timisoara, Romania; bogdananglitoiu@gmail.com (B.A.); patrascujenel@yahoo.com (J.-M.P.J.); sandesc.mihai@umft.ro (M.-A.S.); 2 “Pius Brinzeu” Emergency Clinical County Hospital, Bld Liviu Rebreanu, No. 156, 300723 Timisoara, Romania; alina.abuawwad@umft.ro (S.-A.A.-A.); dinu.anca@umft.ro (A.R.D.); totorean.alina@umft.ro (A.-D.T.); dr_cojocaru@yahoo.com (D.C.); 3 Research Center University Professor Doctor Teodor Șora, Victor Babes University of Medicine and Pharmacy, Eftimie Murgu Square, No. 2, 300041 Timisoara, Romania; 4 Department XII—Discipline of Obstetrics and Gynecology, Victor Babes University of Medicine and Pharmacy, Eftimie Murgu Square, No. 2, 300041 Timisoara, Romania; 5 Department XVI—Balneology, Medical Recovery and Rheumatology, Victor Babes University of Medicine and Pharmacy, Eftimie Murgu Square, No. 2, 300041 Timisoara, Romania; 6 Research Center for Assessment of Human Motion and Functionality and Disability, Victor Babes University of Medicine and Pharmacy, Eftimie Murgu Square, No. 2, 300041 Timisoara, Romania

**Keywords:** staged treatment, posttraumatic tibial osteomyelitis, rib graft, serratus anterior muscle, limb salvage

## Abstract

Osteomyelitis of the tibia is a challenging condition, particularly when it occurs as a result of trauma. This abstract presents a case study detailing the successful staged treatment of posttraumatic tibial osteomyelitis utilizing a unique combination of rib graft and serratus anterior muscle. This medical abstract presents a case study of a 52-year-old male with a history of heavy smoking and obliterating arteriopathy of the lower limbs. The patient sustained a traumatic open fracture classified as Type IIIA Gustilo Anderson involving one-third of the distal right tibia diaphysis, with an associated right fibular malleolus fracture. The treatment approach comprised multiple stages, focusing on wound management, infection control, and limb salvage. The initial stage involved the application of an external fixation device in the emergency setting. Seven days later, an osteosynthesis procedure was performed using a Kuntscher nail and wire cerclage. However, complications emerged, with wound dehiscence and purulent secretion observed at 14 days postsurgery. Subsequently, secondary suturing was carried out at the 20-day mark. The second stage of the treatment involved implant removal, wide excisional debridement, pulse lavage, osteoclasia, and relaxation of the peroneal malleolus. A monoplane external fixation system was applied. As a part of postoperative care, aspiration therapy with a vacuum pump was administered, along with a 10-day course of vancomycin according to the antibiogram. Positive clinical signs of healing were noted, and sterile cultures confirmed the results. The third stage of the intervention focused on grafting the osteo-muscular defect, utilizing autografts from the rib and serratus anterior muscle. The external fixator was maintained in place during this phase. In the fourth and final stage, after an 8-week integration period of the musculocutaneous flap, the external fixator was removed, and internal fixation was accomplished with a blocked Less Invasive Stabilization System (LISS) plate inserted using the Minimally Invasive Plate Osteosynthesis (MIPO) technique. This case underscores the significance of a multistage approach in managing complex limb injuries, emphasizing the importance of timely intervention, infection control, and innovative techniques for limb salvage and restoration of function.

## 1. Introduction

Posttraumatic tibial osteomyelitis is a complex and often refractory condition that presents significant challenges for both patients and healthcare providers. Its genesis is frequently rooted in traumatic injuries, leading to devastating consequences for the affected limb and overall quality of life. In this context, our case report explores a compelling and innovative approach to managing posttraumatic tibial osteomyelitis, centering on the staged treatment with the novel inclusion of rib graft and serratus anterior muscle autografts. This multifaceted case report not only delves into the clinical intricacies of the case but also serves as a testament to the remarkable capabilities of modern orthopedic surgery [1,2,3].

Osteomyelitis, an infection of the bone, represents a formidable challenge in the realm of musculoskeletal health [4,5,6]. When it occurs in the tibia, it can result in substantial morbidity and potentially devastating consequences, necessitating a multifaceted approach to restore function and quality of life. While osteomyelitis can stem from various sources, posttraumatic osteomyelitis, as the name implies, arises following traumatic injuries to the bone. Such injuries often result in open fractures, which increase the risk of infection due to the exposure of the bone to external contaminants. Successful treatment of posttraumatic tibial osteomyelitis requires a delicate balance between infection control, wound management, and strategies to promote limb salvage [1,3,7,8,9].

This case report serves as an invaluable illustration of the critical significance of a staged approach to complex limb injuries, especially when osteomyelitis is involved. It emphasizes the pivotal role of timely intervention and the paramount importance of infection control. Furthermore, this report showcases the innovative use of rib graft and serratus anterior muscle autografts as a viable solution for addressing complex osteo-muscular defects. This case report also underscores the remarkable potential of contemporary orthopedic techniques, like MIPO, to facilitate limb salvage and restore function in the face of formidable challenges. Through a meticulous exploration of this complex case, we aim to contribute to the body of knowledge in the field of orthopedic surgery and inspire further research and innovation in the management of posttraumatic tibial osteomyelitis.

## 2. Case Report

### 2.1. Patient Selection

The subject of this case study was a 52-year-old male patient with a history of chronic heavy smoking and a confirmed diagnosis of obliterating arteriopathy affecting the lower limbs. He presented with a traumatic open fracture of the distal right tibia diaphysis, which was classified as a Type IIIA Gustilo Anderson fracture involving one-third of the tibia’s length. Simultaneously, the patient had suffered a fracture of the right fibular malleolus. Informed consent was obtained from the patient for the entire course of treatment and for the subsequent publication of this case report.

In the presented clinical case, the patient exhibits a notable medical profile, including a daily cigarette consumption of one pack, a lack of surgical history, moderate alcohol intake, and a confirmed diagnosis of peripheral artery disease (PAD) with an absence of other comorbidities.

The patient at the center of this case report is a 52-year-old male who presented with a challenging clinical profile. His medical history includes chronic heavy smoking and a diagnosis of obliterating arteriopathy of the lower limbs, predisposing him to complications in the management of vascular and wound-related issues. The primary injury was a traumatic open fracture of the distal right tibia diaphysis, classified as a Type IIIA Gustilo Anderson fracture, which accounts for a substantial portion of the tibia. This fracture was further complicated by a concurrent right fibular malleolus fracture. The combination of these factors made his case particularly intricate and demanding.

The diagnosis of osteomyelitis was established through a comprehensive approach, considering the patient’s clinical presentation, positive culture findings, and supportive imaging results. The convergence of these diagnostic elements collectively confirmed the presence of osteomyelitis in the patient.

The treatment approach adopted for this patient was a staged one, with distinct phases carefully designed to address the challenges posed by his condition. The initial phase involved the immediate application of an external fixation device in the emergency setting, providing stability to the fractured bone segments. Subsequently, seven days postinjury, an osteosynthesis procedure was undertaken, which included the insertion of a Kuntscher nail and wire cerclage to facilitate the alignment and fixation of the fractured tibia. However, the patient’s course was marked by complications, with wound dehiscence and the emergence of purulent wound secretions observed at the 14-day mark following surgery. In response, a second-stage intervention was initiated, involving implant removal, wide excisional debridement, pulse lavage to cleanse the wound, osteoclasia for bone remodeling, and relaxation of the peroneal malleolus. To maintain bone stability and support the healing process, an external fixation device with a monoplane design was retained.

In addition to these surgical interventions, postoperative care included aspiration therapy with a vacuum pump and a 10-day course of vancomycin, a potent antibiotic agent chosen based on the antibiogram. Positive clinical signs of healing were observed, and sterile cultures confirmed the resolution of the infection.

The third phase of treatment was marked by the unique approach of grafting the osteo-muscular defect with autografts sourced from the rib and serratus anterior muscle. Throughout this stage, the external fixation device continued to provide support and stability.

The fourth and final stage, carried out after an 8-week integration period of the musculocutaneous flap, entailed the removal of the external fixation device and the implementation of internal fixation using a blocked Less Invasive Stabilization System (LISS) plate. This technique, known as Minimally Invasive Plate Osteosynthesis (MIPO), is particularly valuable for reducing the invasiveness of surgical procedures and promoting quicker recovery.

### 2.2. Treatment Staging

**Figure 1:**
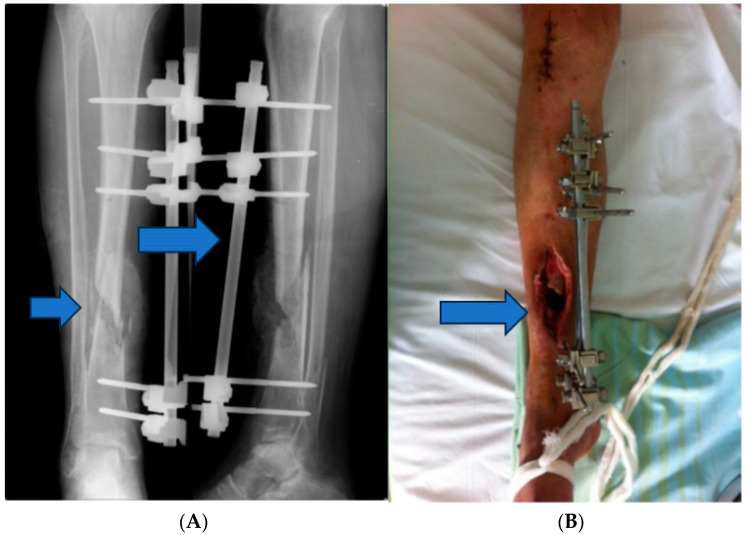
(**A**,**B**) External fixation in the emergency room.

The treatment plan was divided into four distinct stages, each with specific objectives and interventions:

Stage 1: initial stabilization and osteosynthesis (Figure 1).

Objective: To provide immediate stabilization of the open tibial fracture and initiate the process of bone fixation.

Methods: The patient underwent external fixation in the emergency room.

**Figure 2:**
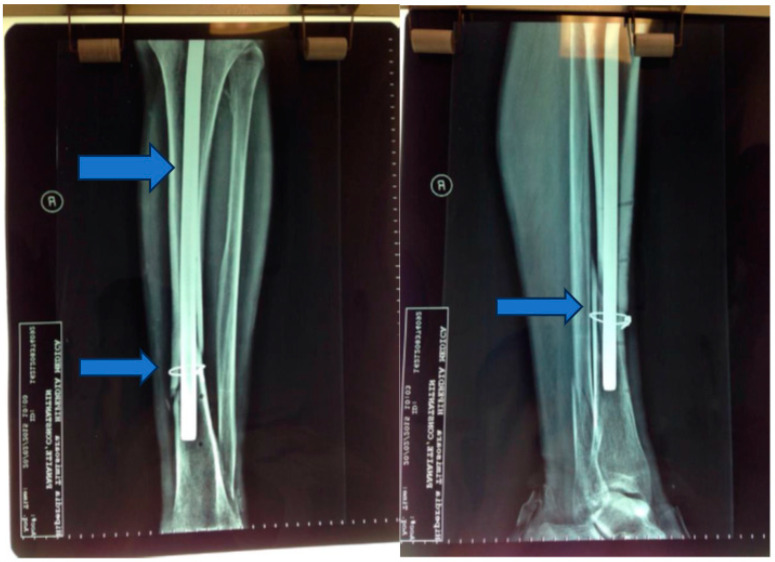
Kuntscher nail and wire cerclage.

**Figure 3:**
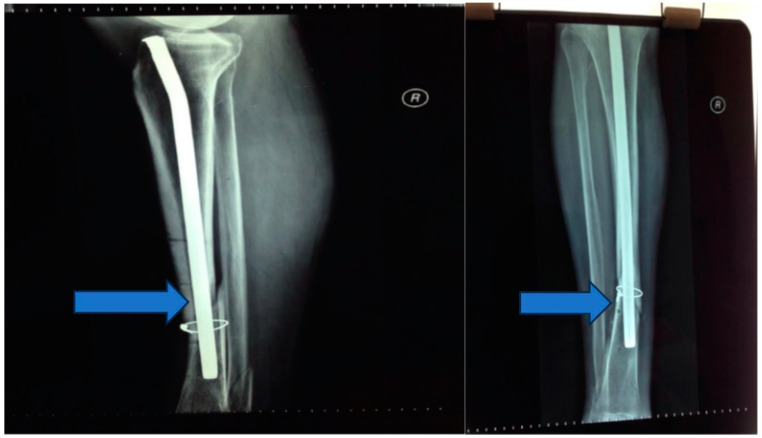
Postoperative X-ray—2 months.

Seven days postinjury, an osteosynthesis procedure was performed, involving the placement of a Kuntscher nail and wire cerclage (Figure 2 and Figure 3).

**Figure 4:**
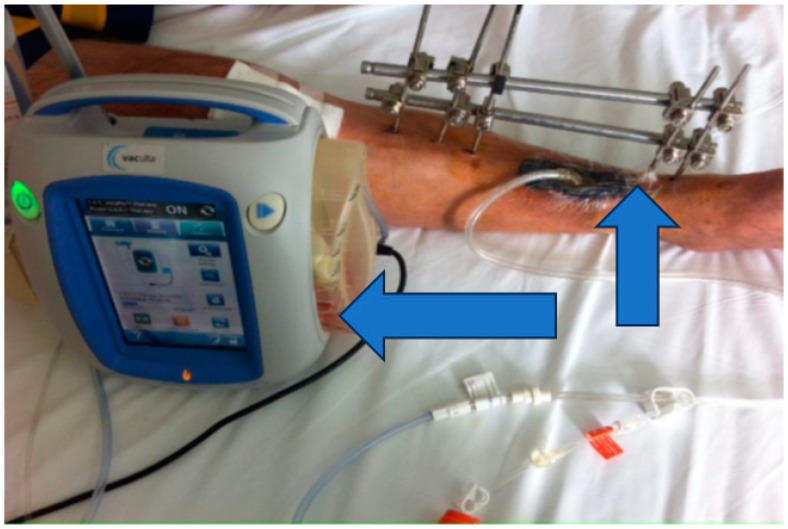
Vacuum pump and ex fix.

Stage 2: Infection control and debridement (Figure 4).

Objective: To address complications, prevent infection, and manage the wound dehiscence.

Methods: Implants from the initial surgery were removed, wide excisional debridement of necrotic tissue was carried out, pulse lavage was performed for wound cleansing, and osteoclasia was executed to facilitate bone healing. The peroneal malleolus was also manipulated, and a monoplane external fixation system was applied. Post-operatively, aspiration therapy using a vacuum pump was employed, and antibiotic therapy with vancomycin (2 g/day) for ten days was initiated based on the results of the antibiogram.

**Figure 5:**
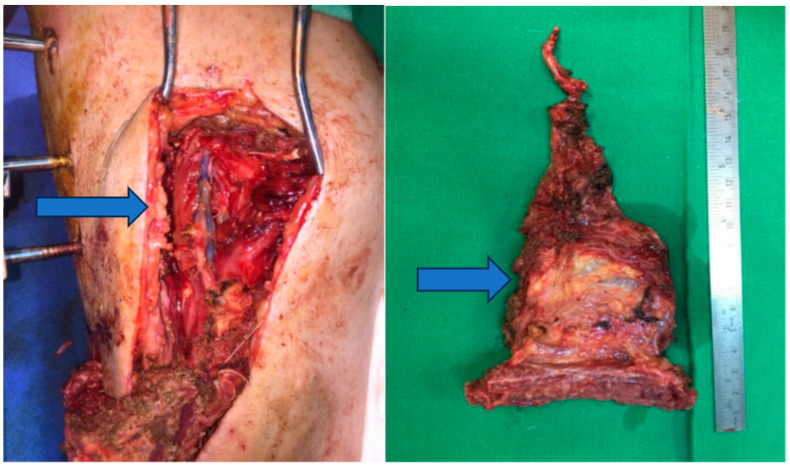
Osteo-muscular defect.

Stage 3: Grafting for osteo-muscular defect (Figure 5).

Objective: To address the osteo-muscular defect and facilitate tissue regeneration.

Methods: This stage involved grafting the osteo-muscular defect using autografts harvested from the rib and serratus anterior muscle. The external fixation device was maintained to provide support during the graft integration period.

**Figure 6:**
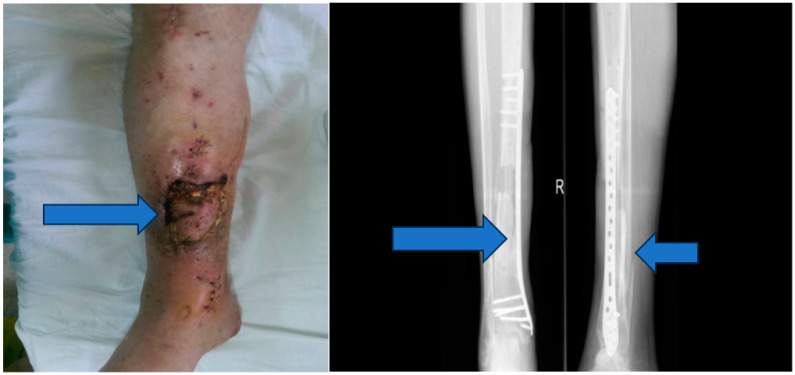
Musculocutaneous flap and internal fixation was achieved using a blocked Less Invasive Stabilization System (LISS) plate.

Stage 4: Flap integration and internal fixation (Figure 6).

Objective: To ensure the integration of the musculocutaneous flap and provide internal fixation to stabilize the tibia.

Methods: After an 8-week integration period, the musculocutaneous flap was deemed stable. The external fixator was removed, and internal fixation was achieved using a blocked Less Invasive Stabilization System (LISS) plate, which was inserted utilizing the Minimally Invasive Plate Osteosynthesis (MIPO) technique.

### 2.3. Antibiotic Therapy and Medication

Antibiotic therapy was initiated as per the results of the antibiogram, with vancomycin administered at a dosage of 2 g/day for ten days postoperatively during the second stage of treatment.

The administration of vancomycin was extended to a duration of 21 days to effectively address the osteomyelitis in accordance with hospital guidelines. This decision was guided by a systematic approach, including negative culture results. The antibiotic regimen was appropriately tailored based on the specific characteristics of the infection and in adherence to established clinical guidelines, ensuring a thorough and evidence-based treatment strategy.

The patient received anticoagulant therapy during the hospitalization period and continued the regimen postdischarge, adhering to the prescribed course for a total duration of 35 days. No alterations were identified in the patient’s everyday medication regimen during the course of our assessment.

### 2.4. Clinical Assessment and Follow-Up

**Figure 7:**
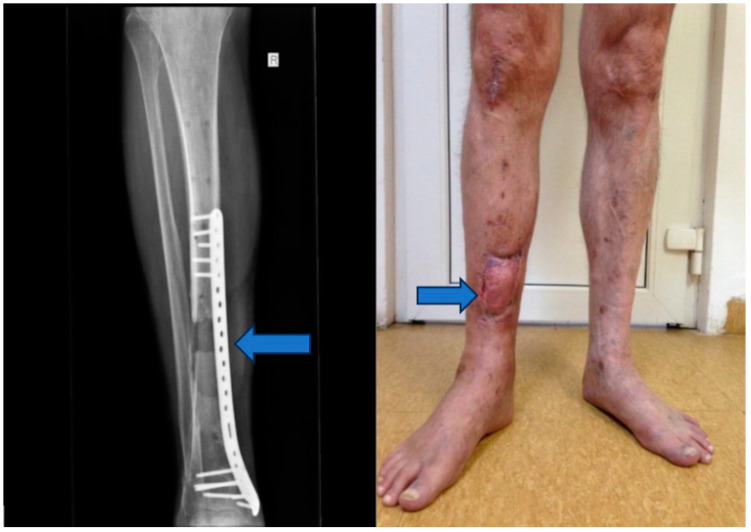
Six-month follow-up.

Throughout the treatment, the patient’s progress was closely monitored, and clinical signs of healing were recorded. Sterile cultures were obtained to verify the resolution of the infection. The patient’s overall condition, pain level, and functional status were assessed at regular intervals (Figure 7).

### 2.5. Data Analysis

Quantitative data, including laboratory results and clinical assessments, were analyzed to evaluate the progress of the patient’s condition throughout the four treatment stages. The success of the staged approach was determined by the resolution of osteomyelitis, the prevention of complications, and the restoration of limb function.

### 2.6. Ethical Considerations

This case study was conducted in accordance with the ethical principles outlined in the Declaration of Helsinki. Informed consent was obtained from the patient for the treatment and subsequent publication of this case report, ensuring the protection of patient confidentiality and privacy.

### 2.7. Management of the Case

The management of posttraumatic tibial osteomyelitis is a complex and challenging endeavor, especially in cases resulting from trauma. This case study details a successful staged treatment approach in a 52-year-old male with a history of heavy smoking and obliterating arteriopathy of the lower limbs who presented with a traumatic open fracture of the right tibia and fibular malleolus. The treatment strategy consisted of multiple stages, focusing on wound management, infection control, and limb salvage.

The initial stage involved the application of an external fixation device in the emergency setting. Subsequent to this, an osteosynthesis procedure using a Kuntscher nail and wire cerclage was performed seven days later. However, complications emerged, with wound dehiscence and purulent secretion observed at 14 days postsurgery. Secondary suturing was undertaken at the 20-day mark, which resolved the wound issues.

The second stage of treatment encompassed implant removal, wide excisional debridement, pulse lavage, osteoclasia, and relaxation of the peroneal malleolus. A monoplane external fixation system was applied, followed by aspiration therapy with a vacuum pump and a 10-day course of vancomycin based on the antibiogram. Positive clinical signs of healing were noted during this phase, and sterile cultures confirmed the absence of infection.

In the third stage, the focus shifted to addressing the osteo-muscular defect through grafting. Autografts from the rib and serratus anterior muscle were utilized to reconstruct the bone and soft tissue. Throughout this stage, the external fixator was maintained to provide stability and support to the graft site.

The fourth and final stage marked the conclusion of the intervention after an 8-week integration period of the musculocutaneous flap. During this phase, the external fixator was removed, and internal fixation was achieved using a blocked Less Invasive Stabilization System (LISS) plate, inserted via the Minimally Invasive Plate Osteosynthesis (MIPO) technique.

This comprehensive approach to managing posttraumatic tibial osteomyelitis led to a successful outcome. The patient experienced significant improvements in clinical symptoms and functional recovery. The staged treatment allowed for the resolution of the infection, restoration of bone continuity, and reconstruction of the soft tissue defect, ultimately leading to limb salvage.

Throughout the entire treatment process, diligent monitoring of the patient’s progress and the use of appropriate antibiotics guided by antibiogram results played a pivotal role in ensuring the success of the intervention.

This case highlights the significance of a multistage approach in managing complex limb injuries, emphasizing the importance of timely intervention, infection control, and innovative techniques for limb salvage and the restoration of function. The utilization of autografts from the rib and serratus anterior muscle represents a novel and effective approach for addressing osteo-muscular defects in such challenging cases. Overall, this case serves as a testament to the potential for successful outcomes in the management of posttraumatic tibial osteomyelitis through a systematic and innovative approach.

## 3. Discussion

Posttraumatic tibial osteomyelitis, especially when resulting from trauma, presents a formidable challenge for healthcare professionals. The case study presented here sheds light on the successful management of this complex condition in a 52-year-old male with a history of heavy smoking and obliterating arteriopathy of the lower limbs. The treatment strategy, involving a multistaged approach, aimed at wound management, infection control, and limb salvage, ultimately led to a positive outcome.

Posttraumatic tibial osteomyelitis poses unique difficulties due to the traumatic etiology, which often results in extensive soft tissue and bone damage [7,8,9,10]. The risk factors associated with this case, such as heavy smoking and arteriopathy, further complicated the treatment. Management strategies must be carefully planned to address these challenges and ensure a successful outcome.

The success of this case can be attributed to the staged treatment approach, which allowed for the systematic and comprehensive management of the condition. Each stage was meticulously planned and executed, with the goals of achieving infection control, promoting healing, and restoring limb function. The use of external fixation, osteosynthesis, debridement, aspiration therapy, and grafting in different stages highlights the importance of tailoring treatment to the specific needs of the patient.

Infection control played a pivotal role in this case, as infection is a common and serious complication of tibial osteomyelitis [11,12,13]. The initial emergence of wound complications was promptly addressed, and secondary suturing was performed to resolve the issues. Subsequently, wide excisional debridement, pulse lavage, and antibiotic therapy based on antibiogram results were employed to eradicate the infection. Monitoring for positive clinical signs of healing and sterile cultures confirmed the success of these interventions.

The innovative use of autografts from the rib and serratus anterior muscle to address the osteo-muscular defect is a noteworthy aspect of this case. This technique showcases the importance of exploring novel approaches to managing complex limb injuries. The combination of bone and soft tissue reconstruction using autografts contributed to the overall success of the treatment, ultimately leading to limb salvage.

The hallmark of this case report is the utilization of innovative techniques in the form of rib graft and serratus anterior muscle autografts. The combination of these grafts allowed for the reconstruction of both bone and soft tissue defects, addressing the complexities of posttraumatic tibial osteomyelitis. This approach exemplifies the necessity of exploring novel methods and tailoring treatment strategies to the specific needs of each patient. The multifaceted approach, consisting of multiple stages and focusing on infection control, wound management, and limb salvage, showcases the versatility and adaptability required to manage such intricate cases successfully.

This case underscores the significance of a multistage approach in the management of complex limb injuries. The timely transition from one stage to the next ensured that the patient received the necessary treatments at the right juncture of their recovery [14,15,16]. This approach minimizes complications and maximizes the chances of successful outcomes.

The staged treatment strategy employed in this case led to significant improvements in the patient’s clinical symptoms and functional recovery. The restoration of bone continuity and reconstruction of soft tissue defects contributed to limb salvage, preserving both function and quality of life for the patient.

Posttraumatic tibial osteomyelitis is a complex and demanding condition that poses significant challenges to both patients and healthcare providers. This case report presented a unique and successful approach to managing this condition through staged treatment involving rib graft and serratus anterior muscle autografts. The intricate interplay of trauma, additional risk factors, and extent of tissue and bone involvement necessitates a comprehensive and innovative treatment strategy. In the culmination of this case, we underscore the significance of such an approach and its potential to enhance patient outcomes and advance the field of orthopedic and trauma surgery.

This case report serves as a testament to the potential for successful outcomes in the management of posttraumatic tibial osteomyelitis through innovative and multifaceted approaches. It is imperative to recognize that while the condition is complex and challenging, there are strategies that can lead to positive results. The patient in this case experienced significant improvements in clinical symptoms and functional recovery, emphasizing the effectiveness of the chosen treatment strategy.

The staged treatment of posttraumatic tibial osteomyelitis with rib graft and serratus anterior muscle is a topic that has gained attention in the recent literature. The existing body of research highlights the significance of adopting a staged approach for managing posttraumatic tibial osteomyelitis, focusing on the utilization of rib grafts and the serratus anterior muscle. Studies underscore the efficacy of this treatment strategy in achieving infection control, promoting healing, and restoring limb function.

Literature reviews indicate that the staged treatment approach allows for systematic and comprehensive management, with each stage meticulously planned to address specific aspects of the condition. The incorporation of rib grafts and the serratus anterior muscle introduces innovative techniques that contribute to the success of the treatment. These techniques not only demonstrate the adaptability of the approach but also underscore the importance of tailoring treatment to the unique needs of each patient.

Overall, the literature supports the notion that the staged treatment of posttraumatic tibial osteomyelitis with rib graft and serratus anterior muscle is a promising avenue for effective and individualized patient care. Further exploration and in-depth analysis of these innovative techniques are warranted to continually enhance our understanding and refine treatment protocols for this challenging condition.

## 4. Conclusions

A key takeaway from this case report is the importance of timely intervention. The staged treatment approach ensured that each aspect of the patient’s condition was addressed at the most opportune moment in their recovery journey. This minimized complications and optimized the chances of a favorable outcome. Additionally, the diligent attention to infection control cannot be overstated. Promptly addressing complications; employing wide excisional debridement, aspiration therapy, and appropriate antibiotic therapy based on antibiogram results; and closely monitoring the patient for positive clinical signs of healing were all critical in eradicating the infection and achieving the ultimate goal of limb salvage.

Moving forward, further research and exploration of similar approaches are paramount. The field of orthopedic and trauma surgery continues to evolve, and innovation is essential to improving outcomes for patients with posttraumatic tibial osteomyelitis. The lessons learned from this case report, particularly the use of rib graft and serratus anterior muscle autografts, could inspire further investigations into the potential benefits of these techniques and the refinement of existing treatment protocols.

In conclusion, the staged treatment of posttraumatic tibial osteomyelitis with rib graft and serratus anterior muscle autografts represents a promising and innovative approach to addressing a challenging medical condition. This case report demonstrates that with a careful and comprehensive strategy, patients can achieve positive outcomes even in the face of complex and multifaceted clinical challenges. As the field of orthopedic and trauma surgery continues to progress, the lessons from this case serve as a reminder of the potential for innovation and improvement in patient care, particularly for those facing the daunting prospect of posttraumatic tibial osteomyelitis.

This case demonstrates the potential for successful outcomes when such an approach is implemented, highlighting the importance of innovative techniques, timely intervention, and meticulous infection control. Further research and exploration of similar approaches are essential to advance the field of orthopedic and trauma surgery and improve the outcomes for patients with posttraumatic tibial osteomyelitis.

## Data Availability

The data presented in this study are available on request from the corresponding author.
